# Economic burden of moderate and severe anxiety and depression symptoms among adults in Saudi Arabia: evidence from a cross-sectional web panel survey

**DOI:** 10.1136/bmjopen-2024-092067

**Published:** 2025-09-26

**Authors:** Karen Arulsamy, Abdulrahman Alfaisal, Jyotika Puri, Mohammed Alluhidan, Yasmin Altwaijri, Abdulhameed Al-Habeeb, Mariam M Hamza, Volkan Cetinkaya, Eric Andrew Finkelstein

**Affiliations:** 1 Duke-NUS Medical School, Singapore; 2 Saudi Health Council, Riyadh, Riyadh, Saudi Arabia; 3 University of Melbourne, Melbourne, Victoria, Australia; 4 King Faisal Specialist Hospital and Research Center, Riyadh, Saudi Arabia; 5 National Center for Mental Health Promotion, Saudi Arabia Ministry of Health, Riyadh, Riyadh, Saudi Arabia; 6 World Bank, Washington, District of Columbia, USA

**Keywords:** MENTAL HEALTH, Health Services, Depression & mood disorders, Anxiety disorders

## Abstract

**Abstract:**

**Objective:**

Anxiety and depression are among the top contributors to disability in the Kingdom of Saudi Arabia (KSA), but little is known about their economic impact. This study estimates the economic burden of moderate to severe symptoms of anxiety and depression among adults in KSA.

**Design and setting:**

A cross-sectional survey was administered via a web panel. Participants were asked to fill out the Patient Health Questionnaire-4 (PHQ-4) for themselves and on behalf of other household members to capture prevalence rates. Those who reported at least moderate symptoms of anxiety or depression filled out a longer survey with questions on healthcare utilisation and productivity losses due to symptoms. These responses were monetised using prevalence rates and population estimates to calculate per-person and total annual costs.

**Participants:**

Prevalence estimates are based on responses from 1164 participants on behalf of 3202 Saudi adults. Of these, 269 individuals with symptoms completed the longer survey.

**Primary outcome measures:**

Prevalence of anxiety and depression; healthcare utilisation (medications, outpatient, inpatient) and productivity losses due to absenteeism and presenteeism.

**Results:**

In total, 26.2% reported at least moderate symptoms consistent with anxiety and/or depression. Among those with symptoms, direct healthcare costs due to anxiety and depression averaged Saudi riyal (SAR) 3431.95 per person annually. Indirect costs via absenteeism and presenteeism averaged SAR 9702.87 and SAR 24 577.28 per person assuming that anxiety and/or depression episodes last for 6 months. Summing up the healthcare costs and productivity losses yields a total annual economic burden of SAR 163.3 billion. Absenteeism accounts for 24.8% of this total (SAR 40.5 billion), presenteeism accounts for 62.8% (SAR 102.5 billion) and healthcare resource utilisation accounts for 12.4%(SAR 20.3 billion).

**Conclusions:**

The overall prevalence of anxiety and depression in KSA is estimated at 26.2%. The economic burden associated with these symptoms amounts to SAR 163.3 billion or 4.1% of GDP. Absenteeism and presenteeism costs account for the vast majority of the total, but a large percentage (nearly 60%) also report emergency department visits and unplanned hospital admissions. Evidence-based interventions should be considered to address the health and economic burden of these conditions in KSA.

**STRENGTHS AND LIMITATIONS OF THIS STUDY**

- Uses a low-cost and expeditious online panel approach.
- Captures both direct healthcare costs and indirect costs due to productivity losses.
- Generalisability may be limited due to the use of an online panel sample.

## Background

 Anxiety and depression are leading causes of disability and premature mortality. Even prior to COVID-19, the WHO ranked depression as the single largest contributor to global disability (7.5% of all years lived with disability in 2015) and anxiety as the sixth (3.4%).^1^ These conditions entail tremendous economic costs including direct healthcare costs and indirect costs incurred in the labour market due to unemployment, absenteeism and presenteeism.^2^ Lost labour market productivity alone is estimated to cost the global economy US$1 trillion per year and is expected to reach US$16 trillion by 2030.^3^

In the Kingdom of Saudi Arabia (KSA), anxiety and depression are ranked among the top six contributors to years lived with disability.^4^ Before COVID-19, the Saudi National Mental Health Survey (SNMHS) showed that 34.2% of individuals aged 15–65 years old had experienced at least one mental health disorder during their lifetime while 20.2% experienced a mental health disorder in the past year. The most commonly reported disorders, based on the WHO Composite International Diagnostic Interview (CIDI), were anxiety disorders (12.3%) and mood disorders (6.8%).^5 6^

Despite the relatively high prevalence of anxiety and depressive disorders among adults in KSA, a dearth of evidence exists on the overall economic burden that these conditions impose. Further, while studies broadly show that the pandemic increased anxiety and depression rates, no recent prevalence estimates are available for KSA.^7^ This study addresses these gaps by estimating the current prevalence and economic burden of diagnosed and undiagnosed moderate to severe anxiety and depression symptoms among adults in KSA.

An innovation of this study is the use of a web panel survey to collect comprehensive information on detailed mental healthcare utilisation and absenteeism and presenteeism rates which are currently unavailable in the SNMHS. Web panels have increased in popularity in recent years as an expedient and low-cost solution to provide timely and credible data to guide resource allocation and policy.^8 9^ Our prevalence estimates are based on the clinically validated Patient Health Questionnaire-4 (PHQ-4).^10^ Our economic burden estimates comprise three key measures: healthcare expenditures based on monetising the value of self-reported mental healthcare utilisation; absenteeism based on the market value of self-reported days missed from work due to anxiety and depression; and presenteeism, defined as the monetised value of self-reported reductions in productivity while working due to anxiety and depression symptoms.

## Methods

### Participants

A cross-sectional online survey was administered to Saudi citizens aged at least 18 years old who are part of a national web panel from a large and established market research firm (Kantar). The Saudi panel comprises 45 000 active panellists from all 13 regions of the country. Participants receive redeemable points for selected rewards in exchange for completing surveys. For this study, Kantar recruited panellists via convenience sampling, sending email invitations to all individuals who met the main inclusion criteria (Saudi citizens aged 18 years and above). Data collection was conducted between September 25 and October 10 2023. The survey was administered either in English or Arabic depending on the respondents’ preference.

To estimate prevalence rates, we first surveyed a broader sample (n=1164), who completed the PHQ-4 for themselves and other adult household members (n=3202). A subset of main respondents who screened positive for anxiety and depression was then invited to complete a longer survey capturing detailed information on healthcare utilisation and labour market productivity. In total, 269 respondents reported at least moderate symptoms of anxiety and/or depression on the PHQ-4 (score ≥6 on the total score) and passed the attention check.

The web panel vendor was asked to ensure a minimum of 100 respondents for the longer survey. This target was informed by prior studies and Kantar’s assessment of feasibility. A sample size of 100 is generally considered sufficient for summarising categorical and continuous treatment variables in descriptive cost-of-illness studies, even when treatment rates in the sample are low. For example, Johnston *et al*^11^ show that a sample size of 100 and an estimated treatment rate of 10% in the sample is expected to provide a 95% CI of ±0.03.^11 12^ We chose to base the sample size on expected treatment rates rather than labour productivity outcomes, as treatment rates were anticipated to be considerably lower. Because we were not comparing outcomes between individuals with and without symptoms, the study was not powered to detect group differences. Kantar ended up surveying 1164 main respondents in the broader sample using the PHQ-4 to meet the target of at least 100 respondents for the longer survey. These respondents provided PHQ-4 information on 3202 adults including themselves. Ultimately, 269 respondents (8.4%) completed the longer survey and passed the attention check. As a result, the response rate of 8.4% out of the total broader sample is an artefact of the study design and reflects the prevalence of reported symptoms, participants’ willingness to complete the longer survey and their ability to pass the attention check. There were no prior comparable web panel studies in KSA to inform the expected response rate. To reduce costs, a targeted recruitment approach was used—rather than inviting all participants, including those without symptoms, to complete the longer survey—since vendor costs were tied to achieving the minimum 100-respondent target.

The full survey is provided in online supplemental material 1). Institutional Review Board (IRB) approval (NUS-IRB-2023–153) was obtained from the National University of Singapore IRB Board on 4 September 2023. Informed consent was obtained from all subjects according to NUS IRB guidelines. All methods were carried out in accordance with this IRB approval.

### Measures

#### Prevalence of anxiety and depression symptoms

Respondents were asked to fill out the PHQ-4 for themselves and on behalf of all other adult household members. The PHQ-4 is a combination of the PHQ-2 and Generalized Anxiety Disorder 2-item (GAD-2), which are validated two-item, 2-week recall period, ultra-brief screeners shown to have high sensitivity (83% and 88%, respectively) and specificity (90% and 82%, respectively) for assessing symptoms of depression and anxiety. The brevity and accuracy of the PHQ-4 enables quick identification of diagnosed and undiagnosed individuals who are likely to present with clinical levels of depression and anxiety symptoms.^10^

Respondents were asked to fill out a longer questionnaire (which takes about 15–20 min to complete) if they reported experiencing symptoms of depression or anxiety. We focused on moderate and severe symptoms of anxiety or depression based on the clinically validated score of ≥6 on the total score (12) of the PHQ-4.^10^ As the respondent also filled out the PHQ-4 on behalf of other adult household members, we obtained PHQ-4 data on a total of 3202 adults. Prevalence rates are calculated by dividing the number of individuals who scored six or more on the PHQ-4 by the total number of individuals across all households in the study.

#### Healthcare utilisation

To quantify costs of healthcare utilisation attributable to symptoms of anxiety and depression, the questionnaire included content on the frequency of visits to healthcare providers, the use of medication and occurrences of serious medical events due to symptoms of anxiety or depression. These questions are based on the Medical Expenditures Panel Survey.^13^ In terms of healthcare visits, information on inperson and telehealth visits to non-specialist providers (eg, government clinic, general practitioner, family medicine and doctor etc), psychiatrists, psychologists and social workers was collected. In terms of medication, information on the frequency (as needed or daily) and duration (less than 1 month, one to 6 months and more than six months) of consumption was collected. With regard to serious medical events, information on whether the respondent visited an emergency department, with or without hospital admission and/or was admitted to the hospital due to symptoms of anxiety and depression and the number of subsequent visits/admissions was collected.

A recall period of 3 months was used for healthcare visits and 12 months for serious medical events. Healthcare visits were multiplied by two to obtain estimated annual visits assuming symptoms last for 6 months out of the year. To monetise healthcare utilisation, unit costs were applied to each type of service based on unsubsidised costs provided by the Saudi Health Council. The full breakdown of unit costs is provided in online supplemental material 2). Per person healthcare cost estimates for adults are obtained by averaging costs across respondents in the sample. Total healthcare cost estimates are calculated by multiplying estimated KSA adult (20 years and older) counts (22.54 million) by the estimated adult prevalence rates and the per person cost estimates.^14^ While our survey was fielded to those aged 18 years old and above, there is no publicly available population count breakdown for those aged 18–19 years old based on census data for us to accurately account for in the estimations (The population count data can be retrieved online here: https://portal.saudicensus.sa/portal/public/1/17/100680?type=TABLE).

#### Absenteeism and presenteeism

For adults, absenteeism and presenteeism costs are calculated based on the human capital approach and accounting for the fact that symptoms of anxiety and depression may not last for a full year.^15^ Specifically, lost productivity is quantified using the Workplace Productivity and Impairment Questionnaire: Specific Health Problem V2 fielded to the subset of respondents who are employed.^16^ The WPAI is commonly used to measure labour productivity losses due to health conditions.^1517^,^19^ In the WPAI, respondents are asked to assess absenteeism and presenteeism in the past 7 days. To calculate absenteeism and presenteeism costs, we assume that each episode of anxiety and/or depression lasts 6 months and occurs within a single year, representing a more conservative approach.

Absenteeism was captured by asking these respondents to indicate the number of hours missed from work due to problems associated with symptoms of depression and/or anxiety in the past 7 days. This figure was then multiplied by 48 (number of weeks in a work-year) to generate annual hours missed in a year and monetised by multiplying by an average hourly wage estimate for each respondent. Hourly wages are calculated by dividing the median monthly income in KSA (Saudi riyal (SAR) 8928.57), based on the SNMHS, by 160.^20^ Monthly income from the general population was used, rather than from our sample so that the market value of lost productivity is more representative of the KSA population. The median value was used, as opposed to the mean, so that results are not skewed by a small subset of very wealthy individuals. The number of days missed from work is multiplied by 0.5, based on an assumption that an individual experiences their symptoms for 6 months, following typical symptomatology and prognosis of these conditions.^21^

Presenteeism is captured based on a question asking employed respondents the degree to which depression and/or anxiety symptoms affected productivity while working in the past 7 days on a scale of 0–10 with 0 being ‘no symptoms and/or symptoms had no effect on my work’ and 10 being ‘symptoms completely prevented me from working’. Monthly presenteeism hours were calculated by multiplying the response on the presenteeism scale/10 by the average weekly number of hours as reported by respondents and scaled up to 48 working weeks. This estimate was then monetised using a similar approach for absenteeism, to calculate per person costs. As only those in the labour force can generate absenteeism and presenteeism costs, we estimated total costs by multiplying the estimated labour force population (15.91 million) with our estimated prevalence rate and per person costs.^14 22^

To limit the effect of outliers, we employed winsorisation at the 95th percentile so that values larger than the 95th percentile are replaced by the 95th percentile. This approach retains the entire sample while circumventing the bias of extreme outliers on key findings.^23^ All results are based on descriptive analyses. There were no missing data on the longer survey, as respondents were required to provide answers through forced-choice questions. Finally, given high comorbidity between symptoms of anxiety and depression, burden estimates for depression and anxiety are combined. All costs are reported in SAR.

#### Patient and public involvement

Patients or the public were not involved in the design, conduct, reporting or dissemination plans of our research.

## Results

### Prevalence of depression and anxiety

Out of 3202 adults, 26.2% had symptoms consistent with anxiety and/or depression, yet 19.5% were never formally diagnosed. The subsequent sections focus on results based on the longer questionnaire and respondents who scored a score of ≥6 on the PHQ-4. After excluding respondents who failed an attention check question, the final sample for analysis includes 269 respondents.

### Respondent characteristics

Table 1 describes characteristics of the main respondents in our sample in comparison to the general population based on official census data.^14^ Compared with the general population, a higher proportion of respondents in our sample are female, married, employed and earn high incomes. Overall, respondents are more advantaged compared with the general population in KSA. Among unemployed respondents (n=28), eight individuals or 28.6% reported that their unemployment was due to their symptoms of anxiety and depression. It is important to note that the sample population includes only respondents who reported at least moderate symptoms of anxiety or depression and completed the longer survey; therefore, systematic differences from the general population are to be expected.

**Table 1:** Characteristics of sample and general population

	Sample population with anxiety and/or depression	General population (based on Census data)
Mean age	33.1 (6.2)	29.0 (NA)
Female (%)	64.7%	38.8%
Married (%)	88.5%	55.0%
Education – tertiary qualification (%)	84.0%	NA
Employed (%)	89.6%	60.4%
Monthly income (%)		
5999 SAR and less than	5.8%	Average monthly income: SAR 10 739
6000 SAR – 14 999 SAR	44.4%	
15 000 SAR and above	49.8%	
EQ-5D-5L overall score	0.747	NA
N	269	32 175 224

Sample includes 269 respondents. Income information only collected from the sample that is employed (n=241 respondents). SD provided in parentheses for continuous variables. The EuroQol 5-Dimension 5-Level (EQ-5D-5L) scores were calculated based on a recently published value set for KSA.^34^ General population characteristics are obtained from the KSA General Authority for Statistics. While the General Authority for Statistics publishes average monthly wages, they do not provide a breakdown that aligns with the income categories collected in our sample. Therefore, we present the average monthly income for employees aged 25–54 years instead.^14^

KSA, Kingdom of Saudi Arabia; SAR, Saudi riyal.

### Annual healthcare resource utilisation

Table 2 provides a detailed breakdown of healthcare utilisation in our sample.

**Table 2:** Healthcare utilisation

	% Used	
Healthcare consultations		Mean (SD) visits per user
Any healthcare consultation in the past 12 months for anxiety/depression	83.3%	–
Any healthcare consultation in the past 3 months for anxiety/depression	79.9%	–
Non-specialist provider (ie, government clinic, General Practitioner, family medicine, doctor, etc)	42.9%	3.91 (2.26)
Psychiatrist	74.6%	4.31 (2.58)
Psychologist	64.3%	3.67 (2.64)
Social worker	40.2%	3.74 (2.48)
Use of medication		
Currently taking any prescription medications to treat anxiety/depression symptoms	91.3%	
1–2 medications	37.6%	
3–5 medications	52.3%	
6 and more medications	10.1%	
Frequency of consumption		
As needed	25.1%	
Daily	74.9%	
Duration of consumption		
Less than 1 month	2.1%	
1–5 months	20.4%	
6 months or more	77.5%	
*Serious medical event*		
Any emergency department (ED) or hospitalisation event in the past 12 months	56.5%	
ED visit without hospitalisation	40.2%	3.11 (2.47)
ED visit with hospitalisation	28.3%	2.97 (2.64)
Direct hospitalisation without an ED event	26.0%	3.13 (2.38)

The sample includes 269 respondents. Questions on healthcare consultation in the past 3 months only asked to respondents who reported that they had seen a healthcare provider in the last 12 months. Mean healthcare visits include tele and inperson visits. Questions on medication use are only asked to respondents who report that they have ever taken any prescription medication (n=218). SD provided in parentheses for continuous variables.

Over 80% visited a healthcare provider in the past 12 months for their mental health symptoms. In the past 3 months, 79.9% visited a healthcare provider; 42.9% consulted a non-specialist provider, 74.6% consulted a psychiatrist, 64.3% consulted a psychologist, and 40.2% consulted a social worker. On average, respondents reported approximately four visits to a healthcare provider in the past 3 months. Respondents also reported a high usage of medication to treat their symptoms, with over 90% currently taking prescription medications. Most respondents take 1–5 medications, take medications daily and have been for more than 6 months.

In the past 12 months, 40.2% of those with these mental health symptoms visited the emergency department, 28.3% visited the emergency department and were subsequently hospitalised and an additional 26% were hospitalised directly. On average, respondents reported approximately three visits to the emergency department or hospital per serious medical event.

### Per person direct and indirect economic burden

Direct healthcare costs due to symptoms of depression and anxiety averaged SAR 3431.95 per patient with these symptoms. These per patient costs are then multiplied by adult population counts and the prevalence rate of 26.2 to obtain overall estimated healthcare costs of SAR 20.3 billion.

Those employed are estimated to miss 21.7 working days on average per person, assuming that symptoms last for 6 months within a year. This equates to SAR 9702.87 in economic losses per person. Respondents reported an average presenteeism score of 6.7, which indicates that these individuals were 67% less productive at work due to their symptoms. This translates to missing an equivalent of 55 working days on average per person, assuming that symptoms last for 6 months within a year. This equates to a loss of SAR 24 577.28 per person. The per person absenteeism and presenteeism costs are each multiplied by the core labour force population and the prevalence rate of 26.2% to obtain overall estimated labour productivity losses of SAR 143 billion.

Table 3 shows the breakdown of per person and total costs by direct and indirect costs and the overall economic burden of anxiety and depression symptoms among adults in KSA. Summing up the health costs and productivity losses yields a total economic burden of depression and anxiety of SAR 163.3 billion. Absenteeism accounts for 24.8% of this total (SAR 40.5 billion), presenteeism accounts for 62.8% (SAR 102.5 billion) and healthcare resource utilisation accounts for 12.4% (SAR 20.3 billion).

**Table 3:** Per person and total costs of depression and anxiety symptoms among adults in KSA

Cost category	Per person costs (SAR)	Total costs (SAR) (BILLION)	Share of costs (%)
Healthcare	3431.95	20.3	12.4 %
Absenteeism	9702.87	40.5	24.8 %
Presenteeism	24 577.28	102.5	62.8 %
**Total**	37 712.10	**163.3**	**100**

Notes: Manual calculations may not sum exactly due to rounding.

KSA, Kingdom of Saudi Arabia; SAR, Saudi riyal.

## Discussion

This is the first study to document the annual economic burden of anxiety and depression symptoms in KSA. Healthcare costs averaged SAR 3431.95 per person with these conditions. Indirect costs due to absenteeism and presenteeism averaged SAR 9702.87 and SAR 24 577.28 per person.

We further showed that 26.2% of adults reported symptoms of anxiety and depression. This prevalence is higher compared with prior findings based on the nationally representative SNMHS which found that, among individuals aged 15–65 years old, 12.3% had experienced anxiety disorders and 6.8% had experienced mood disorders in the past 12 months. These prevalence rates were based on the WHO CIDI which was administered through face-to-face interviews conducted between 2011 and 2016.^5 24^ Unlike the WHO CIDI, the PHQ-4 is not an official diagnostic tool, meaning that not all individuals who report symptoms on it will receive a formal diagnosis. Our prevalence rate is closer to another KSA study based on the PHQ-9 and GAD-7, conducted via online and telephone interviews in 2022, which reported national prevalence rates of 12.7% for MDD and 12.4% for GAD.^25^ Our prevalence rate may also reflect a general rise in anxiety and depression post-COVID 19.^7^ Further, the online nature of our survey likely reduced social desirability bias and increased willingness to disclose potentially stigmatising symptoms.^26^

Approximately 20% of those with anxiety and depression symptoms in our sample had not been formally diagnosed by a healthcare provider. This finding, combined with very high levels of both planned and unplanned (emergency department visits and subsequent admissions) healthcare utilisation, suggests a likely treatment gap. The high per person absenteeism and presenteeism costs further suggest that interventions to prevent or reduce these symptoms have a high chance of being cost-effective or even cost-saving.

Combining the per person cost and prevalence estimates, the economic burden of anxiety and depression symptoms is estimated at SAR 163.3 billion. This is equivalent to 4.1% of KSA’s gross domestic product (GDP) (SAR 4028 billion) in 2023, with labour market productivity losses accounting for over 80% of this burden (International Monetary Fund 2023). A similar study from Singapore found that these symptoms accounted for 2.9% of GDP.^8^ Prior studies from the US estimate the economic burden of diagnosed depression, excluding anxiety, to be 1.6% of GDP.^27^ Estimates of the costs of all mental health conditions, of which depression and anxiety account for the majority, are approximately 4% of GDP in Europe.^28^,^30^ The 4.1% estimate of GDP losses in KSA is higher, reflecting the greater prevalence of mental health symptoms in the sample as well as the associated healthcare utilisation and labour productivity losses.

### Limitations

This study estimates the economic burden of anxiety and depression using a low-cost and expeditious approach. However, there are significant limitations. The main limitation is the reliance on an online panel. Compared with the general population with symptoms, this sample is likely to be more advantaged in terms of educational attainment and income. Therefore, it is possible that individuals in our sample with symptoms of depression and anxiety are not representative of the broader population with these symptoms in KSA. In their analysis of the 12-month prevalence of mental health disorders using SNMHS data, a study found that individuals with the lowest levels of education had significantly lower odds of mood disorders compared with those with the highest levels of education.^5^ No significant associations were found between income and the 12-month prevalence of any class of disorders.^5^ Therefore, as our sample reports higher levels of education, a more representative sample may yield a lower prevalence rate. However, since the SNMHS was conducted between 2011 and 2016, it is also possible that the prevalence rate has increased over time due to other factors that we may be capturing in our data.^31^ We did not collect sufficient demographic information (eg, sex, age, marital status, income and employment) for all respondents and household members reported on by respondents in the larger sample, preventing us from applying weights to the prevalence estimates.

In terms of healthcare utilisation, lower-income respondents with symptoms were less likely to use services compared with those with higher income in the SNMHS. As such, using a more representative sample of those with symptoms may result in lower overall utilisation rates and costs. However, this implication is less clear in our context, as we focus specifically on individuals with moderate to severe symptoms, while the SNMHS includes individuals with a broader range of symptoms.^32^ Limited evidence exists on the role of income and education in influencing the relationship between distress and labour productivity. Based on data from eight countries, those with higher levels of education and higher incomes tend to have lower levels of depression-related absenteeism, whereas higher levels of education are associated with lower absenteeism and higher presenteeism.^33^ Thus, a more representative sample of those with symptoms is likely to increase labour productivity costs.

While the PHQ-4 enables rapid identification of likely clinical symptoms of depression and anxiety, it is not an official diagnostic tool. It is important to note that not everyone who screens positive for symptoms will receive a clinical diagnosis of anxiety or depression. For this reason, we are careful to state that our sample consists of ‘those with symptoms’, not all of whom will have clinical levels of depression and anxiety. Main respondents also filled out the PHQ-4 on behalf of other adult household members, likely introducing reporting bias—it is difficult to predict the direction of this bias. Further, there is a possibility of selection bias where individuals with greater levels of distress and/or mental healthcare use were more likely to respond to the full survey. We also did not capture other costs via other sources of mental healthcare such as digital mental health tools, coaching and medical or diagnostic tests. Specific challenges of conducting research in this context included ensuring accurate Arabic translations, liaising with the web panel vendor to improve data quality and striving to reach a more representative sample. Future studies should aim to corroborate these results using alternative methods, potentially relying on more representative samples and merging data with official medical records to obtain objective measures of healthcare utilisation. Longitudinal studies can also provide evidence on the long-term impact of anxiety and depression on economic outcomes and the effectiveness of prevention and treatment strategies.

## Conclusion

The overall prevalence of anxiety and depression in KSA is estimated at 26.2%. Roughly 20% report not seeking treatment for their symptoms. The economic burden associated with these symptoms amounts to SAR 163.3 billion or 4.1% of GDP. Absenteeism and presenteeism costs account for the vast majority of the total, but a large percentage also report emergency department visits and unplanned hospital admissions. Evidence-based interventions should be considered to address the health and economic burden of these conditions in KSA.

## Supplementary material

10.1136/bmjopen-2024-092067 online supplemental file 1

## Data Availability

Data are available upon reasonable request.
