# Supplementary material for: Economic burden of moderate and severe anxiety and depression symptoms among adults in Saudi Arabia: evidence from a cross-sectional web panel survey
Source: BMJ Open. 2025 Sep 26;15(9):e092067. doi: 10.1136/bmjopen-2024-092067 (PMC12481296; doi:10.1136/bmjopen-2024-092067)
Supplement: online supplemental file 1 [file bmjopen-15-9-s001.docx]

**Supplementary Materials**

**Supplementary Material 1**


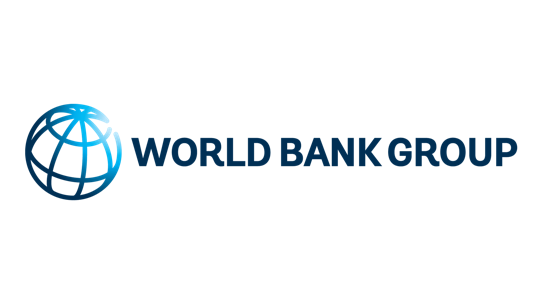

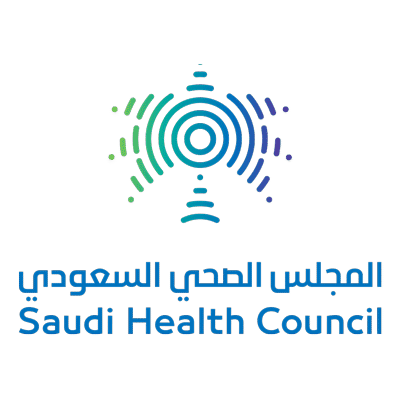


**Saudi Major depressive disorder (MDD) and generalized anxiety disorder (GAD) Survey**

**SCREENER**

**[SC1] Screener Part 1**

S1. Are you a citizen of Saudi Arabia?

| A | Yes |
| --- | --- |
| B | No |

[TERMINATE IF NO]

S2. What is your current age?

|  |
| --- |

[TERMINATE IF < 18]

S3. How many children live in your household between ages 10 and 17?]

[Range 0-10]

| 1 | Zero |
| --- | --- |
| 2 | One |
| 3 | Two |
| 4 | Three |
| 5 | Four |
| 6 | Five |
| 7 | Six |
| 8 | Seven |
| 9 | Eight |
| 10 | If more than eight, please enter number ______ |

[SKIP IF CODED 1 in S3]

S4. Please enter the **ages** of all children living in your household between ages 10 and 17 from oldest age to youngest age:

|  | Enter the age of child [Range10 to 17] |
| --- | --- |
| Child #1 |  |
| Child #2 |  |
| Child #3 |  |
| Child #4 |  |
| Child #5 |  |
| Child #6 |  |
| Child #7 |  |
| Child #8 |  |

*Use additional sheet for additional children, if any.*

S5. How many adult family members (>=18 years of age) live in your household including yourself? [Range 0-10]

| 1 | One |
| --- | --- |
| 2 | Two |
| 3 | Three |
| 4 | Four |
| 5 | Five |
| 6 | Six |
| 7 | Seven |
| 8 | Eight |
| 9 | Nine |
| 10 | Ten |
| 11 | If more than ten, please enter number ______ |

[SKIP IF CODED 1 in S5]

S6. Please enter the **ages** of all adults living in your household from oldest age to youngest age (excluding yourself):

|  | Age (18 or above, maximum of 105) |
| --- | --- |
| Adults #1 |  |
| Adult #2 |  |
| Adult #3 |  |
| Adult #4 |  |
| Adult #5 |  |
| Adult #5 |  |
| Adult #6 |  |
| Adult #7 |  |
| Adult #8 |  |
| Adult #9 |  |
| Adult #10 |  |

**[SC2] SCREENER PART 2 – PHQ-4**

In the next section, we will ask you a series of questions about any symptoms of depression/anxiety that anyone in your household may be actively experiencing. Please consider these questions in the context of the last two weeks.

1. Over the last two weeks, how often have the following individuals been bothered by **feeling nervous, anxious, or on edge**?

|  | Not At all | Several Days | More than half the days | Nearly every day |
| --- | --- | --- | --- | --- |
| Yourself | 0 | 1 | 2 | 3 |
| List out all children by age from oldest to youngest – one per row | 0 | 1 | 2 | 3 |
| List out all adults by age from oldest to youngest – one per row | 0 | 1 | 2 | 3 |

[**to pipe in based on numbers entered in S4 & S6 i.e., the screener items where participants enter the ages of the children and the adults in their household respectively]*

1. Over the last two weeks, how often have the following individuals been bothered by **not being able to stop or control worrying**?

|  | Not At all | Several Days | More than half the days | Nearly every day |
| --- | --- | --- | --- | --- |
| Yourself | 0 | 1 | 2 | 3 |
| List out all children by age from oldest to youngest – one per row | 0 | 1 | 2 | 3 |
| List out all adults by age from oldest to youngest – one per row | 0 | 1 | 2 | 3 |

[**to pipe in based on numbers entered in S4 & S6 i.e., the screener items where participants enter the ages of the children and the adults in their household respectively]*

1. Over the last two weeks, how often have the following individuals been bothered by **little interest or pleasure in doing things**?

|  | Not At all | Several Days | More than half the days | Nearly every day |
| --- | --- | --- | --- | --- |
| Yourself | 0 | 1 | 2 | 3 |
| List out all children by age from oldest to youngest – one per row | 0 | 1 | 2 | 3 |
| List out all adults by age from oldest to youngest – one per row | 0 | 1 | 2 | 3 |

[**to pipe in based on numbers entered in S4 & S6 i.e., the screener items where participants enter the ages of the children and the adults in their household respectively]*

1. Over the last two weeks, how often have the following individuals been bothered by **feeling down, depressed, or hopeless**?

|  | Not At all | Several Days | More than half the days | Nearly every day |
| --- | --- | --- | --- | --- |
| Yourself | 0 | 1 | 2 | 3 |
| List out all children by age from oldest to youngest – one per row | 0 | 1 | 2 | 3 |
| List out all adults by age from oldest to youngest – one per row | 0 | 1 | 2 | 3 |

[**to pipe in based on numbers entered in S4 & S6 i.e., the screener items where participants enter the ages of the children and the adults in their household respectively]*

S7A. Have you EVER been told by a physician that you have depression and/or an anxiety disorder?

| Yes, Depression | Yes, Anxiety Disorder | Yes, both | No |
| --- | --- | --- | --- |
| A | B | C | D |

S7B. Have any of the following **children** **EVER** been told by a physician that they have depression and/or an anxiety disorder?

| List out all children one row at a time from oldest to youngest child | Yes, Depression | Yes, Anxiety Disorder | Yes, both | No |
| --- | --- | --- | --- | --- |
| Child 1 | A | B | C | D |
| Child 2 | A | B | C | D |

S7C. Have any of the following **adults** **EVER** been told by a physician that they have depression and/or an anxiety disorder?

| List out all adults one row at a time from oldest to youngest adult | Yes, Depression | Yes, Anxiety Disorder | Yes, both | No |
| --- | --- | --- | --- | --- |
| Adult 1 | A | B | C | D |
| Adult 2 | A | B | C | D |

Thank you for completing the screener. Your responses indicate that you are eligible to participate in our online survey of individuals with symptoms of depression and/or anxiety. The following questionnaire asks you about your symptoms and their effects on healthcare use and employment**.**

**If you consent to participate, please read through the questions and answer truthfully and to the best of your knowledge. Your answers will be checked thoroughly by the researchers and illogical responses will be flagged.**

**Please be assured that your identity will be kept strictly confidential and only group data will be reported.**

1. I confirm that I consent to participate in this research study.

□ Yes

□ No

[SEEK CONSENT BY ASKING RESPONDENT TO TICK A BOX THAT SAYS: “I confirm that I consent to participate in this study,

[IF participant does not consent, TERMINATE STUDY.]

[IF participant consents, proceed to MHDA1.]

**[MHA1] MAIN SURVEY – MEDICATION HISTORY**

In the next section, you will be asked a series of questions on medications that you have taken or are currently taking to manage your depression/anxiety. We recommend that you have close access to your medications and pill bottles to ensure more accurate responses.

**MHA1.1 Have you EVER taken any prescription medications to treat your depression/anxiety symptoms?**

| **A** | **Yes, for Depression symptoms** |
| --- | --- |
| **B** | **Yes, for Anxiety symptoms** |
| **C** | **Yes, for Both** |
| **D** | **No** |

**[If MHA1.1 = D, SKIP TO HUA1.1]**

**MHA1.2 Are you CURRENTLY taking any prescription medications to treat your depression/anxiety symptoms?**

| **A** | **Yes, for Depression symptoms** |
| --- | --- |
| **B** | **Yes, for Anxiety symptoms** |
| **C** | **Yes, for Both** |
| **D** | **No** |

**[Programming note: If MHA1.2 = D, SKIP TO HUA1.1]**

**MHA1.3 Below is a list of medications that are commonly used to treat symptoms of depression and/or anxiety. Please check Yes for medications that you are CURRENTLY taking and add more if needed**

| **Drug Name** | **No** | **Yes** | **How often do you take this medication?** | | **How long have you been taking this medication?** | | |
| --- | --- | --- | --- | --- | --- | --- | --- |
|  |  |  | **As Needed** | **Once Daily or More** | **<1 Month** | **1 to 6 Months** | **More than 6 Months** |
| Celexa (Citalopram) |  |  |  |  |  |  |  |
| Cymbalta (Duloxetine) |  |  |  |  |  |  |  |
| Effexor (Venlafaxine) |  |  |  |  |  |  |  |
| Lexapro (Escitalopram) |  |  |  |  |  |  |  |
| Luvox (Fluvoxamine) |  |  |  |  |  |  |  |
| Paxil (Paroxetine) |  |  |  |  |  |  |  |
| Prozac (Fluoxetine) |  |  |  |  |  |  |  |
| Zoloft (Sertraline) |  |  |  |  |  |  |  |
| Add medication (Please Specify the brand or generic name of your medication): |  |  |  |  |  |  |  |

**[HUA1] MAIN SURVEY – HEALTHCARE UTILIZATION**

**HUA1.1 In the LAST 12 MONTHS, have you consulted a physician or other healthcare professional regarding your depression/anxiety symptoms?**

| **A** | **Yes** |
| --- | --- |
| **B** | **No** |

**[If HUA1.1 = B, SKIP TO HUA1.3]**

**HUA1.2 In the LAST 3 MONTHS, have you visited (including tele-visits) any of the following healthcare providers *because of your depression/anxiety symptoms*? If yes, please enter the number of visits. For visit types that have more than one healthcare provider listed, select all that apply.**

| **Visit Type** | **No** | **Yes** | **If yes, state the # of tele-visits in the LAST THREE MONTHS.** | **If yes, state the # of in-person visits in the LAST THREE MONTHS** |
| --- | --- | --- | --- | --- |
|  |  |  | **Range for these two columns together cannot be 0** | |
| Non-Specialist Provider (i.e., Government Clinic, General Practitioner, Family Medicine Doctor etc.) |  |  |  |  |
| Psychiatrist |  |  |  |  |
| Psychologist |  |  |  |  |
| Social Worker |  |  |  |  |
| Other (Please specify the type of healthcare provider): |  |  |  |  |

**HUA1.3A In the LAST 12 MONTHS, have you visited an Emergency Department and/or been admitted to the hospital because of your depression/anxiety symptoms?**

| **Visit Type** | **No** | **Yes** | **Specify** |
| --- | --- | --- | --- |
| Emergency department **without** hospital admission |  |  | How many? If yes, [Range 1-50] |
| Emergency department **with** hospital admission |  |  | How many total nights did you spend including all admissions? If yes, [Range 1-50] |
| Direct hospital admission without an ED visit |  |  | How many total nights did you spend including all admissions? If yes, [Range 1-50] |

**[If respondent answers yes to an ED visit with hospital admission and/or direct hospital admission]**

**HUA1.3B Please list the hospital(s) that you were admitted to:**

**HUA1.4 In the LAST 12 MONTHS, have you undergone any medical tests due to your depression/anxiety symptoms?**

| **Test Type** | **Total number of time this test was taken in the last 12 months.** |
| --- | --- |
| 1 | [Range 1-50] |
| 2 | [Range 1-50] |
| 3 | [Range 1-50] |
| 4 | [Range 1-50] |
| 5 | [Range 1-50] |

** No, I have not undergone any medical tests due to my depression/anxiety symptoms.**

**AC1A. What do you think is the world’s current population?**

| **A** | 8 Hundred |
| --- | --- |
| **B** | 8 Thousand |
| **C** | 8 Million |
| **D** | 8 Billion |

**HUA 1.5 How likely are you to consult a physician or other healthcare professional regarding your depression/ anxiety symptoms in the next 3 MONTHS?**

| **A** | Very Likely |
| --- | --- |
| **B** | Somewhat Likely |
| **C** | Not So Likely |
| **D** | Not At All Likely |

**[WPAI-A] MAIN SURVEY – ABSENTEEISM AND PRESENTEEISM (WPAI)**

**WPAI-A1. Are you currently employed or self-employed (i.e., working for pay)?**

| **A** | **Employed** |
| --- | --- |
| **B** | **Self Employed** |
| **C** | **Unemployed** |

**[If WPAI-A1= C, then show only WPAI-A2 and WPAI-A7 from this section]**

**WPAI-A2. Are you currently not working because of your depression/anxiety symptoms?**

| **A** | **Yes** |
| --- | --- |
| **B** | **No** |

**[If WPAI-A1= A or B]**

**The following questions ask about the effect of your mental health-related symptoms on your ability to work over the LAST SEVEN DAYS, not including today. Please fill in the blanks as indicated.**

**WPAI-A3. During the LAST SEVEN DAYS, how many hours did you miss from work because of your depression/anxiety symptoms?** *Include hours missed on days you did not work at all, days you went in late, days you left early, etc. because of your symptoms. Do not include time missed to participate in this study.*

____________Hours [Range 1-98 hours]

**WPAI-A4. During the LAST SEVEN DAYS, how many hours did you miss from work because of any other reasons, such as vacation, holidays, time off to participate in this study?**

____________Hours [Range 1-98 hours]

**WPAI-A5. During the LAST SEVEN DAYS, how many hours did you actually work? [ Please deduct the hours you missed from work because of your depression/anxiety symptoms and hours missed for reasons such as vacation etc.]**

____________Hours [Range 1-98 hours]

**WPAI-A6. During the LAST SEVEN DAYS, how much did your depression/anxiety symptoms affect your productivity while you were working?** *Think about days you were limited in the amount or kind of work you could do, days when you accomplished less than you would like, or days you could not do your work as carefully as usual. If your symptoms affected your work only a little, choose a low number. Choose a high number if your symptoms affected your work a great deal.*

| No symptoms and/or symptoms had no effect on my work | 0 | 1 | 2 | 3 | 4 | 5 | 6 | 7 | 8 | 9 | 10 | Symptoms completely prevented me from working |
| --- | --- | --- | --- | --- | --- | --- | --- | --- | --- | --- | --- | --- |

**WPAI-A7. During the LAST SEVEN DAYS, how much did your depression/anxiety symptoms affect your ability to do your regular daily activities, other than work at a job?** *By regular activities, we mean the usual activities you do such as work around the house, shopping, childcare, studying. Think about times you were limited in the amount or kind of activities you could do and times you accomplished less than you would like. If your mental health symptoms affected your activities only a little, choose a low number. Choose a high number if your mental health symptoms affected your activities a great deal.*

| No symptoms and/or symptoms had no effect on my daily activities | 0 | 1 | 2 | 3 | 4 | 5 | 6 | 7 | 8 | 9 | 10 | Symptoms completely prevented me from doing my daily activities |
| --- | --- | --- | --- | --- | --- | --- | --- | --- | --- | --- | --- | --- |

**WPAI-A8. What is your typical monthly employment income from all sources (including self-employed/business owner)?**

| **1** | **Less than SAR 3000** |
| --- | --- |
| **2** | **SAR 3000 – SAR 5999** |
| **3** | **SAR 6000 – SAR 8999** |
| **4** | **SAR 9000 – SAR 11999** |
| **5** | **SAR 12000 – SAR 14999** |
| **6** | **SAR 15000 – SAR 17999** |
| **7** | **SAR 18000 – SAR 20999** |
| **8** | **SAR 21000 – SAR 23999** |
| **9** | **SAR 24,000 and over** |
| **99** | **Prefer not to answer** |

**[EQ5D-5L-A] MAIN SURVEY – QUALITY OF LIFE ASSESSMENT (EQ5D-5L)**

Under each heading, please tick the ONE box that best describes your health TODAY.

**EQ5D-5L-A1. Mobility**

|  | I have no problems in walking about |
| --- | --- |
|  | I have slight problems in walking about |
|  | I have moderate problems in walking about |
|  | I have severe problems in walking about |
|  | I am unable to walk out |

**EQ5D-5L-A2. Self-Care**

|  | I have no problems washing or dressing myself |
| --- | --- |
|  | I have slight problems washing or dressing myself |
|  | I have moderate problems washing or dressing myself |
|  | I have severe problems washing or dressing myself |
|  | I am unable to wash or dress myself |

**EQ5D-5L-A3. Usual Activities (e.g., work, study, housework, family or leisure activities)**

|  | I have no problems doing my usual activities |
| --- | --- |
|  | I have slight problems doing my usual activities |
|  | I have moderate problems doing my usual activities |
|  | I have severe problems doing my usual activities |
|  | I am unable to do my usual activities |

**EQ5D-5L-A4. Pain/Discomfort**

|  | I have no pain or discomfort |
| --- | --- |
|  | I have slight pain or discomfort |
|  | I have moderate pain or discomfort |
|  | I have severe pain or discomfort |
|  | I have extreme pain or discomfort |

**EQ5D-5L-A5. Anxiety/Depression**

|  | I am not anxious or depressed |
| --- | --- |
|  | I am slightly anxious or depressed |
|  | I am moderately anxious or depressed |
|  | I am severely anxious or depressed |
|  | I am extremely anxious or depressed |

**
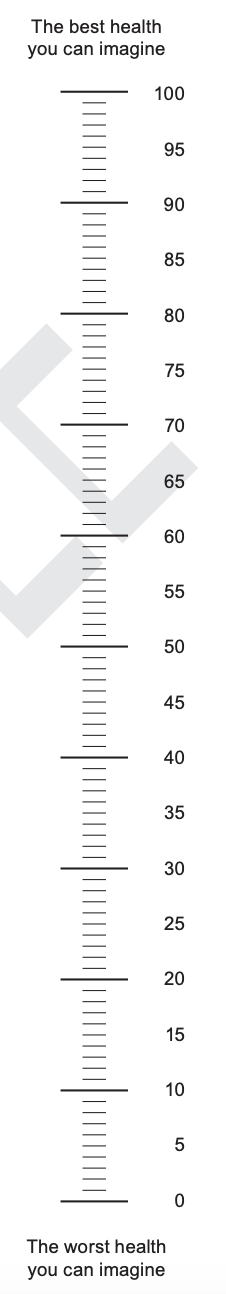
EQ-5D-ASCALE**

We would like to know how good or bad your health is TODAY.

- This scale is numbered from 0 to 100.
- 100 means the best health you can imagine. 0 means the worst health you can imagine.
- Mark an X on the scale to indicate how your health is TODAY.
- Now, please write the number you marked on the scale here: _______

**[PSC1.6A] INTEREST IN USING PEER SUPPORT FOR MENTAL HEALTH**

**PSA1.1 Who do you speak with about the challenges in your life? (check all that apply)**

| **A** | Family members |
| --- | --- |
| **B** | Friends |
| **C** | Colleagues |
| **D** | Psychologist |
| **E** | Life coach |
| **F** | Spiritual or religious leader |
| **G** | Other (specify) |
| **H** | I do not speak with anyone about my challenges |

**Definition of Peer Support**: Peer support refers to informal and non-professional support from someone who shares a similar background or experience (e.g., same mental health diagnosis and/or treatment experience). The support may be both informational and emotional and may be individual or in groups. During these sessions, individuals share their experiences living with their condition and its impact on themselves and their family. Peer support is intended to be used in non-crisis situations and is not meant to replace professional treatment.

**PSA1.2 Have you ever participated in peer support for mental health as a mentor or mentee?**

| **A** | Yes, Mentor |
| --- | --- |
| **B** | Yes, Mentee |
| **C** | No |

**PSA1.3 How interested would you be in utilizing peer support for your depression/anxiety symptoms if it were free of charge?**

| **A** | Very Interested |
| --- | --- |
| **B** | Somewhat Interested |
| **C** | Not So Interested |
| **D** | Not At All Interested |

**PSA1.4 Do you see peer support for mental health as a substitute (something to try instead of seeking professional care from a formal healthcare provider) or complement (something to do alongside) to professional mental health support?**

| **A** | Peer support as a substitute to professional mental health care |
| --- | --- |
| **B** | Peer support as a complement to professional mental health care |
| **C** | Don’t know/Unsure |

**PSA1.5 Would you be willing to provide your personal information (eg, name, contact information) to a peer supporter if required for participation?**

| **A** | Yes |
| --- | --- |
| **B** | No |

[If PSA.1.3 = A or B] Ask the following set of questions.

**PSA1.6A Please check which of the below types of peer support you would be most interested in.**

| **A** | One-on-one |
| --- | --- |
| **B** | Small Group |
| **C** | No preference |

**PSA1.6B Please check which of the below modes of delivery you would be most interested in.**

| **A** | Virtual (tele- or web-based) |
| --- | --- |
| **B** | In person |
| **C** | No preference |

**PSA1.6C What would you be looking to get out of a peer support program? Check all that apply**

| **____** | Connection with someone who has had a similar experience |
| --- | --- |
| **____** | Emotional support- someone to listen and provide encouragement |
| **____** | Information and knowledge about your condition/ treatments |
| **____** | To learn strategies to cope with your condition |
| **____** | Other (Specify): |

[If PSC.1.3 = C or D] Ask the following question.

**PSA1.7 Why does peer support not appeal to you? Please check all that apply.**

| **A** | I have concerns about confidentiality |
| --- | --- |
| **B** | I do not think it will help me |
| **C** | I think peer support will worsen my mental health |
| **D** | It is too time consuming |
| **E** | I suspect the quality will be low |
| **F** | It is likely to be too expensive |
| **G** | I do not like talking about my problems to a non-professional |
| **H** | Other (Specify): ________________________________ |

**[SSDSA1] Self-Stigma of Depression Scale Items**

Please indicate the degree to which you agree or disagree with the following statements.

SSDSA1.1 If I had depression or anxiety, I would feel embarrassed about seeking professional help for it.

| 1 | Strongly Disagree |
| --- | --- |
| 2 | Disagree |
| 3 | Agree & Disagree Equally |
| 4 | Agree |
| 5 | Strongly Agree |

SSDSA1.2 If I had depression or anxiety, I would feel embarrassed if others knew I was seeking professional help for it.

| 1 | Strongly Disagree |
| --- | --- |
| 2 | Disagree |
| 3 | Agree & Disagree Equally |
| 4 | Agree |
| 5 | Strongly Agree |

SSDSA1.3 If I had depression or anxiety, I would see myself as weak if I took antidepressants or anti-anxiety medication.

| 1 | Strongly Disagree |
| --- | --- |
| 2 | Disagree |
| 3 | Agree & Disagree Equally |
| 4 | Agree |
| 5 | Strongly Agree |

SSDSA1.4 If I had depression or anxiety, I wouldn’t want people to know that I wasn’t coping.

| 1 | Strongly Disagree |
| --- | --- |
| 2 | Disagree |
| 3 | Agree & Disagree Equally |
| 4 | Agree |
| 5 | Strongly Agree |

**[DSA1] OTHER DEMOGRAPHIC INFORMATION**

**DSA1.1Please record your gender?**

| **1** | **Male** |
| --- | --- |
| **2** | **Female** |

**DSA1.2 What is your current marital status?**

| **1** | **Married** |
| --- | --- |
| **2** | **Separated** |
| **3** | **Widowed** |
| **4** | **Divorced** |
| **5** | **Single** |

**DSA1.3 What is your highest education level completed?**

| **1** | **No Formal Education** |
| --- | --- |
| **2** | **Primary** |
| **3** | **Preparatory/Middle** |
| **4** | **Technical School** |
| **5** | **Secondary/High School** |
| **6** | **University** |
| **7** | **Post-graduate studies** |

**DSA1.4 Which category best describes your occupation?**

| **1** | Student |  |
| --- | --- | --- |
|  |  | Undergraduate |
|  |  | Graduate |
|  |  | Other, please specify |
| **2** | Employee | Government |
|  |  | Private sector |
|  | Military | Junior |
|  |  | Senior |
| **3** | Health worker | Physician |
|  |  | Nurse |
|  |  | Pharmacist |
|  |  | Laboratory technician |
|  |  | Other |
| **4** | Professional (e.g. accountant, engineer etc. with little/no managerial capacity) |  |
|  |  |  |
| **5** | Craftsman or Related Trade Worker (e.g. electrician, plumber, foreman) |  |
| **6** | Self-employed |  |
| **7** | Other, please specify: |  |

Thank you for taking part in the survey.

**Supplementary Material 2**

The unit costs below were sourced based on price lists from Alhabib and Alhamadi Hospitals and provided by the Saudi Health Council.

| Type | Average unit costs (SAR) |
| --- | --- |
| Medication – Daily | 60.3 |
| Medication – As needed | 2.01 a day for one medication |
| Non-specialist consultation | 50 |
| Psychiatrist consultation | 250 |
| Psychologist consultation | 250 |
| Social worker consultation | 250 |
| Emergency department visit | 75 |
| Emergency department visit and hospital admission | 250 |
| Hospital admission | 250 |
