# Supplementary material for: Economic burden of moderate and severe anxiety and depression symptoms among adults in Saudi Arabia: evidence from a cross-sectional web panel survey
Source: BMJ Open. 2025 Sep 26;15(9):e092067. doi: 10.1136/bmjopen-2024-092067 (PMC12481296; doi:10.1136/bmjopen-2024-092067)
Supplement: Supplementary file 2 [file bmjopen-2024-092067.reviewer_comments.pdf]

PEER REVIEW HISTORY

BMJ Open publishes all reviews undertaken for accepted manuscripts. Reviewers are asked to complete a checklist review form and are provided with free text boxes to elaborate on their assessment. These free text comments are reproduced below.

ARTICLE DETAILS

Title (Provisional)

The economic burden of moderate and severe anxiety and depression symptoms among adults in Saudi Arabia: Evidence from a cross-sectional web panel survey

Authors

Arulsamy, Karen; Alfaisal, Abdulrahman; Puri, Jyotika; Alluhidan, Mohammed; Altwaijri, Yasmin; Al-Habeeb, Abdulhameed; Hamza, Mariam M.; Cetinkaya, Volkan; Finkelstein, Eric Andrew

VERSION 1 - REVIEW

**Reviewer**	**1**
**Name**	**Bauer, Annette**
**Affiliation**	**London School of Economics and Political Science**
**Date**	**09-Feb-2025**
**COI**	**None**

This is an important and overall well written study that seeks to provide an estimate of the costs of depression and anxiety symptoms in Saudi Arabia. This research is original and important as it might help raise awareness of the size of the problem of mental health problems in a country where there is currently little evidence.

Cost studies can use different methodologies and it is therefore important to be very transparent about the limitations and assumptions of such studies.

One main concern is that the cost estimates are reliant on a self-report from a highly selective, skewed population, and authors currently do not sufficiently consider the implications of the sample for the findings. The authors should be cautious about inferring transferability from the web based panel survey to the whole population since the group invited to survey is not representative of the overall population but also the response rate to the survey seems to have been low (please clarify the numbers). Some of this is recognized but overall there is not enough information and explanation. It would be useful to have some more information about the challenges of conducting the research in the specific context, and learnings from it for future research. The authors need to provide more information about how individuals were recruited into the study and how missing data were

treated. There is no information about what kind of statistical analysis was conducted if any. It is important to also be clear about the limitations of establishing diagnoses via a survey.

I wonder if it was overall more helpful to present this study as exploratory because of the challenges presented and adapt the title and main text accordingly.

Minor:

1) Table 1:

-The Anxiety/ Depression heading does not make sense; it seems that this should be replaced with sample population;

-Clarify what is meant by 'main respondents' and explain the sample size of n=255

-Why are there no data available on income for the general population?

2) Clearly define what is captured under healthcare expenditure and why; at the moment it sometimes says mental healthcare and other times healthcare; were all serious medical events and emergency visits included, even if unrelated to mental health?

**Reviewer**	**2**
**Name**	**Moinuddin, Mohammed**
**Affiliation**	**University of Lancashire, School of Medicine and Dentistry**
**Date**	**10-Mar-2025**
**COI**	**None**

Statistical reviewer’s comment

The authors presented the results from a cross-sectional study estimating the economic burden of the symptoms of anxiety and depression in KSA. A valuable piece of research however there is a major issue in calculating the prevalence of anxiety and depression prevalence which has a direct implication on the cost estimation. My specific observations are listed below:

Comment 1 – In the method section it is mentioned that 255 respondents filled out the entire survey however in the abstract, the initial number is more than two thousand. Can the author describe the full information in the method? For example, how many were invited? What was the response rate in the initial screening and how many were finally included?

Comment 2 – “… ultra-brief screeners shown to have high sensitivity (83 percent and 88 percent respectively) and specificity (90 percent and 82 percent respectively) for assessing symptoms of depression and anxiety.” Can the author please provide the reference for these data?

Comment 3 – The authors used a higher cut-off (a score of 4 or more) while the standard cut-off is 3 or more. Does this make the screening process less sensitive and give a higher false negative rate? Also, the data of sensitivity and specificity were referred to in the previous section which I guess is based on the standard cut-off. How would the authors justify using the higher cut-off value? Also, using this cut-off the results state that the prevalence of anxiety and depression is 23.9% (19.4% anxiety, 16.4% depression). I believe, if standard cut-off were used the prevalence would be much higher. In a recent study in KSA, it was reported the prevalence of depressive and anxiety disorders is 12.7 and 12.4 percent (Alhabeeb et al, 2023). Therefore, I doubt that the current study represents the KSA population because the prevalence seems much higher even with a higher cut-off diagnosis criterion. The results therefore could be inflated systematically.

Comment 4 – Table 1 shows that the study sample is very different from the general population in terms of sex, marital status, and employment. However, the authors calculated the total number of people with anxiety and depression just by simple multiplication. Also, the absenteeism, presenteeism and cost were estimated in the same way. That means the authors assumed that the rate of anxiety/depression in this sample is similar in the population which of course is not the case. Therefore, it can be argued that the estimated economic burden is much inflated. The potential solution can be to apply sapling weight to estimate the corrected prevalence of anxiety/depression that represents the population values. At least three stratification factors need to be considered in calculating the sample weight: sex, marital status and employment status.

Comment 5 – For calculating the absenteeism, the authors used a recall period of one week. Would this be a valid measure based on one week of recall? Can the authors provide a reference in favour of this choice? Also, please specify what recall period was used for calculating the presenteeism.

Comment 6 – In Table 3, the number is also to be presented with a percentage.

Comment 4 – page 6 or 31, line 7- inconsistent in-text citation style.

References

Alhabeeb, A. A., Al-Duraihem, R. A., Alasmary, S., Alkhamaali, Z., Althumiri, N. A., & BinDhim, N. F. (2023). National screening for anxiety and depression in Saudi Arabia 2022. Frontiers in public health, 11, 1213851. https://doi.org/10.3389/fpubh.2023.1213851

VERSION 1 - AUTHOR RESPONSE

Reviewer 1

The reviewer notes 'This is an important and overall well written study that seeks to provide an estimate of the costs of depression and anxiety symptoms in Saudi Arabia. This research is

original and important as it might help raise awareness of the size of the problem of mental health problems in a country where there is currently little evidence.'.

Cost studies can use different methodologies and it is therefore important to be very transparent about the limitations and assumptions of such studies. One main concern is that the cost estimates are reliant on a self-report from a highly selective, skewed population, and authors currently do not sufficiently consider the implications of the sample for the findings. The authors should be cautious about inferring transferability from the web based panel survey to the whole population since the group invited to survey is not representative of the overall population but also the response rate to the survey seems to have been low (please clarify the numbers). Some of this is recognized but overall there is not enough information and explanation.

The authors need to provide more information about how individuals were recruited into the study and how missing data were treated.

It is important to note that the sample population described in Table 1 includes only respondents who reported at least moderate symptoms of anxiety and depression and completed the longer survey; therefore, systematic differences from the general population are to be expected. We have included a sentence on page 5 (paragraph 4) to clarify this.

However, we acknowledge that there is evidence to suggest that online panels may skew towards higher income respondents. We have now provided a more detailed discussion on potential implications of this on our results in paragraph 2 of page 9 informed by findings from SNMHS.

While 2,828 respondents completed the PHQ-4 on behalf of themselves and adult household members, we had requested that the web panel vendor provide at least 100 main respondents for the longer survey. Thus, the final sample size for the longer survey is not a result of low response rates, but rather an artefact of the study design.

Information on how participants were recruited is provided on page 2 paragraph 5.

We had no missing data as respondents were required to provide answers through forced-choice questions. This information has been added on page 5 paragraph 2.

It would be useful to have some more information about the challenges of conducting the research in the specific context, and learnings from it for future research.

We have added more information on challenges encountered during the study in the Discussion section page 9 (paragraph 3).

There is no information about what kind of statistical analysis was conducted if any.

The results we present are based on descriptive analyses. We have amended paragraph 2 on page 5 to clarify this.

It is important to also be clear about the limitations of establishing diagnoses via a survey.

We have provided more detail in paragraph 3 of page 9 to discuss the limitations of diagnoses via a survey.

Minor:
1) Table 1:
-The Anxiety/ Depression heading does not make sense; it seems that this should be replaced with sample population;

We have amended Table 1 (page 5) accordingly. It is important to note that this table compares the sample population with at least moderate symptoms of anxiety and depression who responded to the longer questionnaire (N=103) which is a subset of the overall sample which filled out the initial part of the questionnaire consisting of the PHQ-4 on behalf of themselves and other household members (N=2,828).

-Clarify what is meant by 'main respondents' and explain the sample size of n=255

In the manuscript, we refer to 'main respondents' (N=103) as those who reported at least moderate symptoms of anxiety or depression based on the PHQ-4 and filled out the longer survey with questions on mental healthcare utilization and labour productivity losses due to symptoms. We also excluded respondents who did not pass the attention check question. We have included an explanation in paragraph 3 (page 5) to clarify what 'main respondents' mean and how the sample size of 103 is derived.

-Why are there no data available on income for the general population?

While the KSA General Authority for Statistics publishes average monthly wages, they do not provide a breakdown that is comparable to the income breakdown collected in our sample. To address this comment, we have now provided the average monthly income based on data published for Q4 2023 and included an explanation in table notes. Changes are highlighted in Table 1 (page 5).

2) Clearly define what is captured under healthcare expenditure and why; at the moment it sometimes says mental healthcare and other times healthcare; were all serious medical events and emergency visits included, even if unrelated to mental health?

Healthcare expenditure includes costs related to medications, outpatient visits, and serious medical events (such as hospitalizations and emergency department visits) incurred specifically due to symptoms of anxiety or depression and will cover healthcare more broadly including mental healthcare.

We have revised paragraph 5 on page 3 to clearly define what is captured under healthcare costs. We have also replaced the use of 'mental healthcare' with 'healthcare' to avoid confusion.

Reviewer: 2

Comment 1 – In the method section it is mentioned that 255 respondents filled out the entire survey however in the abstract, the initial number is more than two thousand. Can the author

describe the full information in the method? For example, how many were invited? What was the response rate in the initial screening and how many were finally included?

In order to estimate prevalence rates, we needed to survey a broader sample (N=2828). Responses from a smaller sample of main respondents (N=103) who reported at least moderate symptoms of anxiety and depression, based on a score of 6 and above on the PHQ-4, were used to assess healthcare utilization and labour market productivity.

While 2,828 respondents completed the PHQ-4 on behalf of themselves and adult household members, we had requested that the web panel vendor provide at least 100 main respondents for the longer survey. Thus, the final sample size for the longer survey (N = 103 for PHQ-4 score of ≥6); N=255 for PHQ-4 score of ≥4) is not a result of low response rates, but rather an artefact of the study design.

We have provided a more detailed explanation of the design of the study in page 2 paragraph 6 onwards.

Comment 2 – "… ultra-brief screeners shown to have high sensitivity (83 percent and 88 percent respectively) and specificity (90 percent and 82 percent respectively) for assessing symptoms of depression and anxiety." Can the author please provide the reference for these data?

We have included the references in paragraph 3 of page 3.

Comment 3 – The authors used a higher cut-off (a score of 4 or more) while the standard cut-off is 3 or more. Does this make the screening process less sensitive and give a higher false negative rate? Also, the data of sensitivity and specificity were referred to in the previous section which I guess is based on the standard cut-off. How would the authors justify using the higher cut-off value? Also, using this cut-off the results state that the prevalence of anxiety and depression is 23.9% (19.4% anxiety, 16.4% depression). I believe, if standard cut-off were used the prevalence would be much higher. In a recent study in KSA, it was reported the prevalence of depressive and anxiety disorders is 12.7 and 12.4 percent (Alhabeeb et al, 2023). Therefore, I doubt that the current study represents the KSA population because the prevalence seems much higher even with a higher cut-off diagnosis criterion. The results therefore could be inflated systematically.

Thank you for raising this point. We used a higher cut-off to better capture more serious clinical symptoms. We acknowledge that the cut-off score of ≥4 has not been clinically validated. To ensure we are capturing probable clinical symptoms, we are now using a higher cut-off score of ≥6, which corresponds to at least moderate symptoms of anxiety/depression. While this reduces the sample size for the main analysis and significantly lowers the overall economic estimates, the resulting prevalence rate (7.3%) is now more consistent with prior studies conducted in KSA.

Comment 4 – Table 1 shows that the study sample is very different from the general population in terms of sex, marital status, and employment. However, the authors calculated the total number of people with anxiety and depression just by simple multiplication. Also, the absenteeism, presenteeism and cost were estimated in the same way. That means the authors

assumed that the rate of anxiety/depression in this sample is similar in the population which of course is not the case. Therefore, it can be argued that the estimated economic burden is much inflated. The potential solution can be to apply sapling weight to estimate the corrected prevalence of anxiety/depression that represents the population values. At least three stratification factors need to be considered in calculating the sample weight: sex, marital status and employment status.

It is important to note that the sample population described in Table 1 includes only respondents who reported at least moderate symptoms of anxiety and depression and completed the longer survey; therefore, systematic differences from the general population are to be expected. We have included a sentence on page 5 (paragraph 4) to clarify this.

With the revised cut-off point, the prevalence rates are now more consistent with previous studies conducted in KSA. With the revised cut-off point, the prevalence rates are now more consistent with previous studies conducted in KSA. While the main sample is now more aligned with the general population in terms of key characteristics, it remains difficult to determine whether differences between the sample and general population reflects a skewed sample or inherent differences within the population experiencing anxiety and depression.

While we acknowledge that weighting would have been ideal, it is not possible to weight the data in this case as detailed demographic information was only collected from respondents who completed the longer version of the survey (i.e. those who reported symptoms N=103), not those who only answered the initial section containing the PHQ-4 (N=2,828).

Comment 5 – For calculating the absenteeism, the authors used a recall period of one week. Would this be a valid measure based on one week of recall? Can the authors provide a reference in favour of this choice? Also, please specify what recall period was used for calculating the presenteeism.

We used a recall period of seven days for the absenteeism and presenteeism questions following the validated Workplace Productivity and Impairment Questionnaire: Specific Health Problem V2 (1). The WPAI and accompanying recall period of seven days is commonly used in studies assessing labour productivity losses due to health conditions (2-5).

We have made revisions on paragraph 2 to 4 on page 4 to make the recall period clearer and provide evidence supporting their use.

Comment 6 – In Table 3, the number is also to be presented with a percentage.

We have revised the table accordingly.

Comment 4 – page 6 or 31, line 7- inconsistent in-text citation style.

We have revised the citation style accordingly.

Further changes:

We have revised text and results throughout the manuscript to reflect changes due to using a higher cut-off point on the PHQ-4.

We have provided more information on how the presenteeism burden estimates are calculated (page 4 paragraph 4).

We have provided more information on how healthcare utilization costs are calculated (page 3 paragraph 6).

References:

1. Reilly MC, Zbrozek AS, Dukes EM. The validity and reproducibility of a work productivity and activity impairment instrument. PharmacoEconomics. 1993;4:353-65.
2. Zhang W, Tocher P, L'Heureux J, Sou J, Sun H. Measuring, analyzing, and presenting work productivity loss in randomized controlled trials: a scoping review. Value in Health. 2023;26(1):123-37.
3. Tang K. Estimating productivity costs in health economic evaluations: a review of instruments and psychometric evidence. PharmacoEconomics. 2015;33(1):31-48.
4. Kigozi J, Jowett S, Lewis M, Barton P, Coast J. The Estimation and Inclusion of Presenteeism Costs in Applied Economic Evaluation: A Systematic Review. Value in Health. 2017;20(3):496-506.
5. Krol M, Brouwer W. How to estimate productivity costs in economic evaluations. PharmacoEconomics. 2014;32:335-44.

VERSION 2 - REVIEW

**Reviewer**	**2**
**Name**	**Moinuddin, Mohammed**
**Affiliation**	**University of Lancashire, School of Medicine and Dentistry**
**Date**	**04-Sep-2025**
**COI**	

I thank the authors for submitting a revised version with a revised analysis and clarification. However, a couple of points require further attention.

The sample data were very different from the general population (Table 1) in terms of sex, age, marital status, etc. Please acknowledge this as a limitation. The authors can add a subheading, 'limitations, ' at the end of the Discussion section, where these issues can be clearly acknowledged. Specifically, there is a lack of representativeness of the sample.

Second, it is difficult to understand why weighting is not possible when population-level data on sex, marital status, and employment are available. Please clarify why this is not possible. In addition, this should be discussed in the limitations section. If the authors do not want to change their analysis by applying weighting, they can present the weighted analysis as a sensitivity analysis.

VERSION 2 - AUTHOR RESPONSE

Reviewer: 2
Dr. Mohammed Moinuddin, University of Lancashire
Comments to the Author:
I thank the authors for submitting a revised version with a revised analysis and clarification. However, a couple of points require further attention.

The sample data were very different from the general population (Table 1) in terms of sex, age, marital status, etc. Please acknowledge this as a limitation. The authors can add a subheading, 'limitations, ' at the end of the Discussion section, where these issues can be clearly acknowledged. Specifically, there is a lack of representativeness of the sample.

Yes, differences in the sample compared to the general population is expected as the sample captures individuals with symptoms of anxiety and depression within a web panel sample and who were also willing and/or ended up responding to the longer survey.

We have now included a 'Limitations' subheading under the Discussion section to more clearly signpost the limitations of the study. The first paragraph under the Limitations subheading highlights this specific limitation (i.e., as web panel samples typically skew towards more advantaged groups, our sample of individuals with anxiety and depression may not be representative of the broader population with these symptoms in KSA) and we have revised the relevant sentence to make this limitation clearer.

Second, it is difficult to understand why weighting is not possible when population-level data on sex, marital status, and employment are available. Please clarify why this is not possible. In addition, this should be discussed in the limitations section. If the authors do not want to change their analysis by applying weighting, they can present the weighted analysis as a sensitivity analysis.

Although population-level data on these variables exist, they were not collected in the first part of the survey for all respondents and household members, during which respondents report the PHQ-4 for themselves and other household members to facilitate re-weighting of prevalence rates. Consequently, post-stratification weights cannot be applied to these variables, as they are absent from our dataset.

We collected these variables in the longer part of the survey, which included questions on healthcare utilization and labour productivity. While the Saudi National Mental Health Survey (SNMHS) also collects these variables, there is no publicly available information detailed enough to inform post-stratification weights, as this would require knowledge of the breakdown of these variables specifically for individuals with at least moderate symptoms of anxiety or depression based on the PHQ-4. It is also important to note that the longer survey has a smaller sample size, which may result in some categories within these variables having very few respondents, potentially biasing the weighting process. Nevertheless, in the Limitations sub-section of the Discussion, we address how changes in demographic characteristics may affect our results in relation to healthcare utilization and labour productivity losses.

We have also revised the sentence at the end of the first paragraph under the Limitations sub-section to explain more clearly why we could not weight the prevalence rates.

We hope this has clarified the points raised but please let us know if any further information is needed.
